# Hysterectomy and the risk of urinary incontinence: a systematic review and meta-analysis

**DOI:** 10.3389/fruro.2026.1816956

**Published:** 2026-06-04

**Authors:** Liping Wang, Leilei Gao, Xuechai Bai, Juan Gu

**Affiliations:** 1 Center for General Practice Medicine, Zhejiang Provincial People’s Hospital/Affiliated People’s Hospital, Hangzhou Medical College, Hangzhou, Zhejiang, China; 2 Department of Nursing, Zhejiang Provincial People’s Hospital/Affiliated People’s Hospital, Hangzhou Medical College, Hangzhou, Zhejiang, China; 3 Center for Reproductive Medicine, Zhejiang Provincial People’s Hospital/Affiliated People’s Hospital, Hangzhou Medical College, Hangzhou, Zhejiang, China

**Keywords:** abdominal hysterectomy, hysterectomy, laparoscopy hysterectomy, meta-analysis, urinary incontinence, vaginal hysterectomy

## Abstract

**Object:**

To evaluate the association between hysterectomy and the risk of developing urinary incontinence (UI) based on observational studies.

**Methods:**

We conducted a systematic search of PubMed, Embase, and Cochrane Library for observational studies from inception to December 14, 2025, using medical subject headings (MeSH) and keywords. The risk of bias and the quality of evidence were assessed using the Newcastle-Ottawa Scale (NOS), the Agency for Healthcare Research and Quality (AHRQ) criteria, and the Grading of Recommendations Assessment, Development, and Evaluation (GRADE) system, respectively. To derive overall summary estimates of odds ratios (OR), a random-effects meta-analysis was performed, complemented by subgroup analyses to explore potential effect modifiers. And the presence of publication bias was evaluated through funnel plots and Egger’s regression test.

**Results:**

This meta-analysis is registered with PROSPERO (CRD42024587774) and follows PRISMA guidelines, including 12 studies with a cumulative total of 146,759 individuals who underwent hysterectomy. The pooled analysis revealed a significant association between hysterectomy and an increased risk of UI, yielding an OR of 1.31, (95% CI: 1.03-1.66, I^2^ = 88.5%, *P* = 0.029). Subgroup analyses showed that the risk of UI was notably higher among patients who underwent abdominal hysterectomy (OR = 1.21, 95% CI: 1.10-1.34, I^2^ = 0.0%, *P* = 0.000). Furthermore, it was observed that the incidence of UI was particularly elevated in studies conducted in Asia, while no significant association was reported for regions such as Europe and North America.

**Conclusion:**

Hysterectomy is associated with an increased risk of urinary incontinence, based on synthesized evidence from observational studies.

**Systematic review registration:**

https://www.crd.york.ac.uk/prospero/, identifier CRD42024587774.

## 1 Introduction

Hysterectomy is one of the most prevalent surgical procedures in gynecology, ranking as the second most common operation globally, following cesarean section. This surgery provides a definitive treatment option for various benign conditions, including cervical lesions, endometrial lesions, uterine fibroids, and adenomyosis (1, 2). Among the different surgical techniques, laparoscopic hysterectomy (LH) stands out as the most frequently performed method, accounting for 59% of cases, followed by vaginal hysterectomy (VH) at 25% and abdominal hysterectomy (AH) at 17% (3, 4). While VH is typically recommended as the first-line approach, LH has increasingly supplanted AH in clinical practice due to its minimally invasive nature and associated benefits (5, 6).

Despite the effectiveness of hysterectomy in treating benign conditions, it has been recognized to disrupt the pelvic floor’s anatomy and supportive structures, potentially leading to significant structural and functional alterations. These changes may manifest as alterations in bladder and bowel functions, as well as sexual dysfunction and dyspareunia (7–9). One of the most significant long-term complications associated with hysterectomy is urinary incontinence (UI), a condition that can severely affect an individual’s quality of life. UI is identified as one of the five major health issues confronting individuals today, contributing to psychological conditions such as depression and loneliness, while also imposing considerable social and financial burdens on healthcare systems. The most prevalent subtype of UI is stress urinary incontinence (SUI), which accounts for approximately 45.9% of cases, followed by urgency urinary incontinence at 31.1% and mixed urinary incontinence at 18.1% (10).

A considerable body of research has examined the relationship between hysterectomy and the risk of developing UI, particularly SUI. Many studies indicate an association between hysterectomy and an increased risk of urinary incontinence, with heightened odds reported among women aged 60 years or older (11, 12). However, these findings are not entirely consistent, as some studies have shown no significant association between hysterectomy and the increased risk of UI (3, 13). To better elucidate the relationship between hysterectomy and the risk of urinary incontinence, we conducted a systematic review and meta-analysis of existing evidence derived from comprehensive observational studies.

## 2 Methods

This study was conducted in accordance with the guidelines set forth by the Preferred Reporting Items for Systematic Reviews and Meta-Analyses (PRISMA) (14). The research protocol was pre-registered on the International Prospective Register of Systematic Reviews (PROSPERO) platform, with the approval number CRD42024587774.

### 2.1 Data sources

We conducted a systematic search of PubMed, Embase, and the Cochrane Library for studies published from the inception of these databases until December 14, 2025. The search strategy utilized both Medical Subject Headings (MeSH) and relevant keywords, including “hysterectomy”, “hysterectomy*”, “urinary incontinence”, and their variants. Details of the search strategy can be found in supplementary **Supplementary Table 1–S3**.

### 2.2 Eligibility criteria

We included observational studies based on the following inclusion criteria: (1) observational design; (2) investigation of the association between hysterectomy and the risk of UI.

Exclusion criteria included: (1) studies not reporting odds ratios (OR) with corresponding 95% confidence intervals (CIs); (2) unavailable full texts; (3) conference abstracts, study protocols, letters to the editor, and studies lacking outcomes of interest.

### 2.3 Study selection

Two reviewers (WLP and GJ) independently performed the study selection based on the established inclusion and exclusion criteria. Initially, they excluded duplicates and irrelevant articles based on titles and abstracts. Subsequently, they retrieved and reviewed the full texts of potentially eligible articles to identify all qualifying studies. Any disagreements were resolved through consultation with a third reviewer (GLL).

### 2.4 Data extraction

Data extraction was performed independently by the two reviewers in accordance with established guidelines for systematic reviews and meta-analyses. We extracted the following data: first author, year of publication, sample size, age, study region, and study type. Discrepancies were resolved through discussion with BXC to reach a consensus.

### 2.5 Risk of bias

The quality of the included studies was assessed using the Newcastle-Ottawa Scale (NOS), which evaluates three key aspects: selection, comparability, and exposure (15). Studies were assigned a star rating from 0 to 9, with a higher number of stars indicating superior study quality. Scores of 0-3, 4-6, and 7-9 were categorized as low, moderate, and high quality, respectively.

For cross-sectional studies, we utilized the Agency for Healthcare Research and Quality (AHRQ) criteria to assess quality (16). Scores of 0-3, 4-7, and 8-11 were classified as low, medium, and high quality, respectively.

### 2.6 Evidence certainty

The overall certainty of the evidence was evaluated using the Grading of Recommendations Assessment, Development, and Evaluation (GRADE) system (17, 18). According to the GRADE system, evidence derived from observational studies starts with a low-quality rating, with potential gradations of high, moderate, low, or very low for specific outcomes.

### 2.7 Statistical analysis

Urinary incontinence was analyzed as a dichotomous outcome, and the odds ratio (OR) was selected *a priori* as the common effect measure (19). This choice was based on standard meta-analytic guidance for dichotomous outcomes, for which ORs and risk ratios are commonly used relative effect measures. Ratio measures are typically analyzed on the logarithmic scale; therefore, reported ORs and 95% confidence intervals (CIs) were transformed into log ORs and corresponding standard errors for pooling. The pooled estimates were then back-transformed and presented as ORs with 95% CIs. We did not combine different effect measures as if they were directly interchangeable; studies without extractable ORs or sufficient information for conversion were excluded from the quantitative synthesis. Heterogeneity was assessed using the χ^2^ test and I^2^ statistic. A fixed-effect model was used when heterogeneity was low (P ≥ 0.10 and I^2^ < 50%); otherwise, a random-effects model was applied. Sensitivity analysis was conducted to assess the robustness of the pooled estimates. Publication bias was evaluated by visual inspection of funnel plots and Egger’s regression test. Subgroup analyses were performed according to urinary incontinence subtype, geographical region, and surgical route. All statistical analyses were conducted using Stata version 14.0.

## 3 Results

### 3.1 Literature search

**Figure 1:**
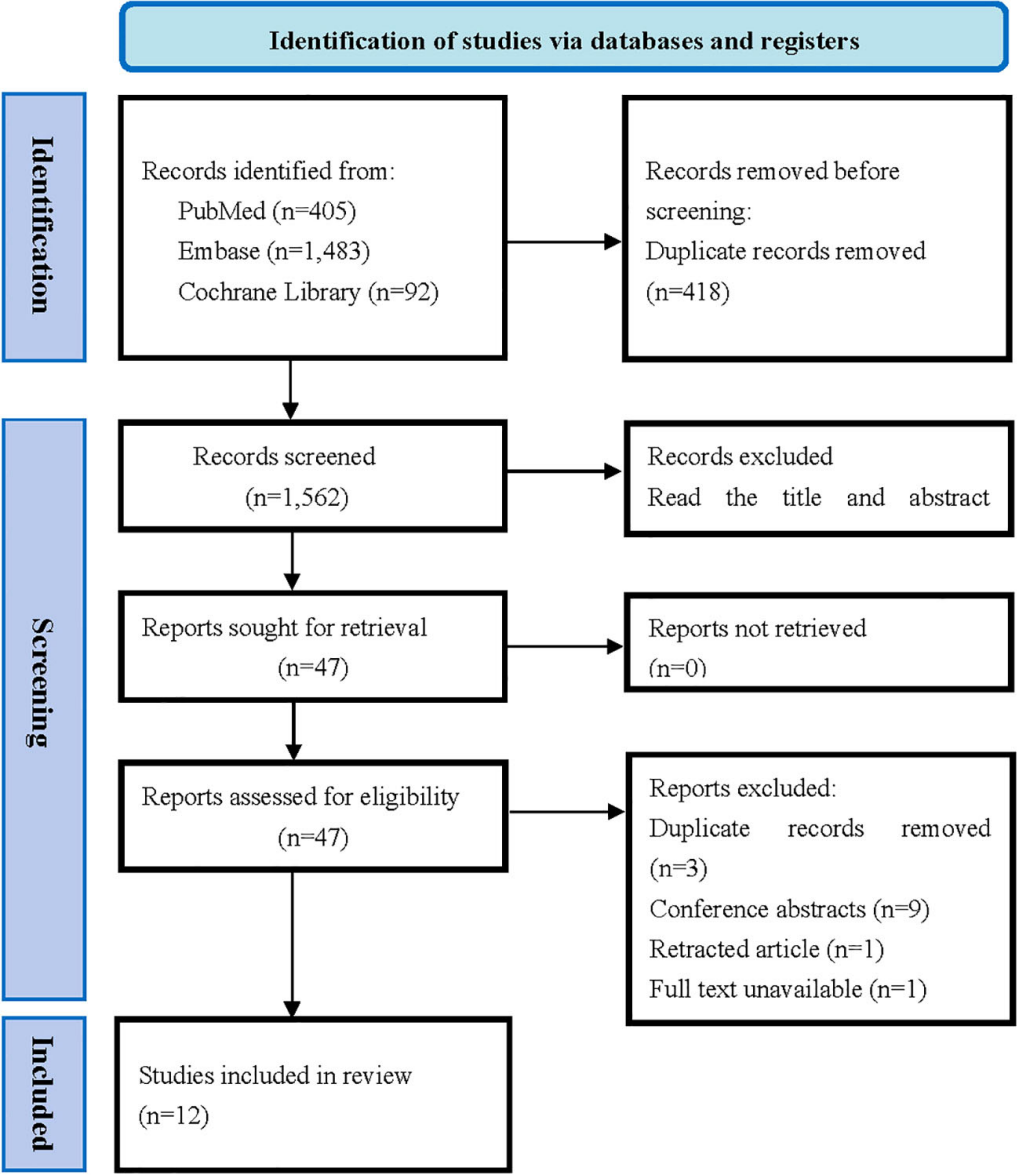
The selection process.

A total of 1,980 records were identified through the search. After screening titles and abstracts, 47 articles were deemed potentially relevant. Following full-text review, 12 studies reporting UI incidence were included. The selection process is illustrated in **Figure 1**.

### 3.2 Study characteristics

This meta-analysis encompassed 12 studies involving 146,759 individuals who underwent hysterectomy, published up to December 14, 2025 (3, 11, 20–29). Detailed characteristics of the included studies are presented in **Table 1**, while excluded studies are listed in **Supplementary Table 4**.

**Table 1:** Characteristics of the included studies.

Author	Year	Country	Study period	Study type	Case group	Control group	Control type	Age (Mean ± SD)	Effect value type
Salo H	2024	Finland	1968 to 2020	A cohort study	425	3495	No hysterectomy	NA	OR
Yuk JS	2023	South Korea	2007 to 2020	A cohort study	81,373	81,373	No hysterectomy	46 (50-43)	OR
Li PC	2019	China	2000 to 2012	A cohort study	8514	34,056	No hysterectomy	47.1	OR
Juliato CR	2017	Brazilian	2012 to 2013	Across-sectional study	111	638	No Previous hysterectomy	52.5 ± 4.4	OR
Linde JM	2017	Netherlands	NA	A cross-sectional study	678	NA	No hysterectomy	48.7 ± 15.3	OR
Kudish BI	2014	The US	NA	A cohort study	38,524	53,569	Uterus in place	50-79	OR
Kirss F	2013	Tallinn	1999 to 2004	Long-term preventive trial	1823	NA	No hysterectomy background	53.3 ± 4.0	OR
Barghouti FF	2013	Jordan	2009 to 2010	A cross-sectional study	73	928	No hysterectomy	46.6	OR
Byles J	2009	Australian	1996 to 2005	A cohort study	12,432	NA	No hysterectomy	70-75	OR
Ham E	2009	Korea	NA	A case-control study	63	545	No hysterectomy	48 ± 5.77	OR
Miller JJ	2008	The US	2003 to 2006	A case-control study	166	166	No hysterectomy	54.8 (25-80)	OR
Minassian VA	2008	The US	2001 to 2002	A case-control study	2,577	NA	No hysterectomy	51	OR

NA, not application.

### 3.3 Quality assessment

Using the NOS criteria, the average score for the included observational studies was 5.11, with all studies scoring 4 or above, indicating moderate to high quality. Additionally, three cross-sectional studies were assessed, yielding an average score of 3.67, as detailed in **Supplementary Tables 5–S6**.

### 3.4 Risk of UI in participants undergoing hysterectomy

Of the 12 included articles, 3 studies only reported specific subgroup results (20, 23, 29), and nine studies reported the association between hysterectomy and UI risk (3, 11, 16, 22, 24–28). The pooled analysis revealed a significant association, with an odds ratio (OR) of 1.31 (95% CI 1.03-1.66, I^2^=88.5%, *P* = 0.029, N = 9), supported by robust sensitivity analyses (results in **Supplementary Table 7**, **Supplementary Figure 1**).

**Figure 2:**
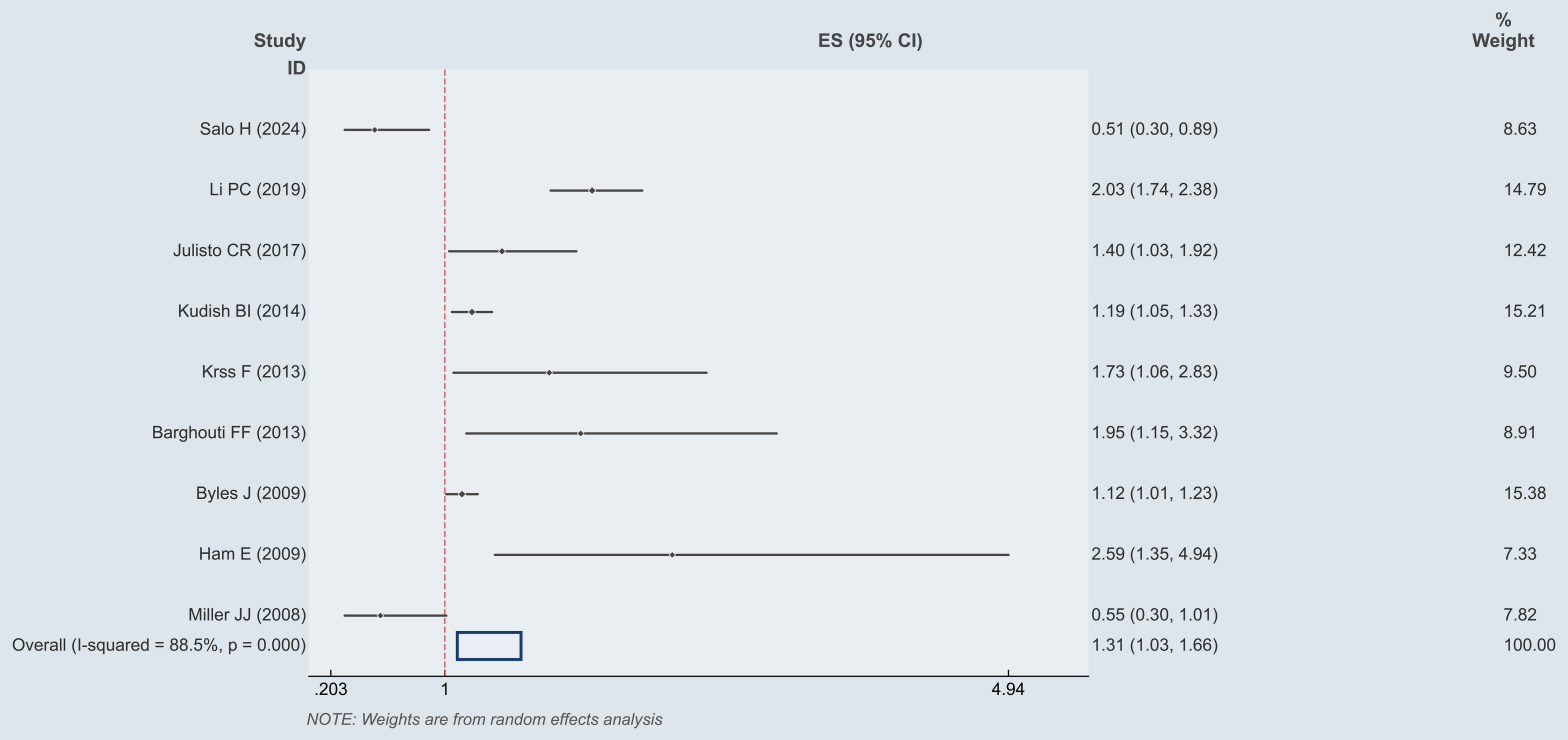
Forest plot for odds of UI in participants underwent hysterectomy.

Given the substantial heterogeneity observed in the primary analysis, exploratory univariable meta-regression was performed to examine whether selected study-level characteristics contributed to between-study variability. As shown in **Table 2**, publication year was not significantly associated with the effect estimate (coefficient = -0.011, 95% CI: -0.101 to 0.078, P = 0.773). Similarly, neither log-transformed sample size (coefficient = 0.020, 95% CI: -0.198 to 0.237, P = 0.836) nor mean/median age (coefficient = -0.024, 95% CI: -0.059 to 0.011, P = 0.150) significantly explained the observed heterogeneity. These findings suggest that the substantial heterogeneity was unlikely to be attributable to a single study-level factor and may instead reflect combined clinical and methodological differences across studies (**Figure 2**).

**Table 2:** Exploratory univariable meta-regression for potential sources of heterogeneity.

Covariate	No. of studies	Coefficient	SE	95% CI	P value
Publication year	9	-0.011	0.038	-0.101 to 0.078	0.773
Sample size, log-transformed	9	0.02	0.092	-0.198 to 0.237	0.836
Mean/median age	8	-0.024	0.014	-0.059 to 0.011	0.150

### 3.5 Subgroup analysis

Subgroup analyses indicated a slight increase in UI risk across stress urinary incontinence, urge urinary incontinence, and mixed urinary incontinence (**Table 3**). A significantly elevated risk of UI was observed in individuals undergoing abdominal hysterectomy; however, no increased risk was found in those undergoing vaginal or laparoscopic hysterectomy. Additionally, hysterectomy was associated with a markedly significant risk of UI in Asia, while no such risk was observed in Europe and North America (**Table 3**). Subgroup analyses suggested that the association between hysterectomy and urinary incontinence varied across regions and surgical routes. A higher risk was observed in studies conducted in Asia, whereas no statistically significant association was detected in studies from Europe or North America. However, these subgroup findings should be interpreted cautiously because each subgroup included a limited number of studies, and regional differences in patient characteristics, clinical practice, surgical indications, follow-up duration, and reporting of urinary incontinence may have influenced the estimates. Therefore, these findings should be considered exploratory rather than confirmatory.

**Table 3:** The subgroup analysis for UI in participants underwent hysterectomy.

Subgroups	Included studies	OR (95% CI)	Heterogeneity	
			*I ^2^* (%)	*P*-value
Type of urinary incontinence				
Stress urinary incontinence	5	1.10 (0.94, 1.21)	55.2	0.290
Urge urinary incontinence	2	1.21 (1.00, 1.46)	11.1	0.050
Mixed urinary incontinence	2	1.57 (0.90, 2.74)	76.3	0.108
Type of hysterectomy				
Abdominal hysterectomy	3	1.21 (1.10, 1.34)	0.0	0.000
Vaginal hysterectomy	2	1.24 (0.34, 4.54)	81.7	0.744
Laparoscopy hysterectomy	2	0.70 (0.27, 1.84)	82.1	0.468
Region				
Asia	3	2.05 (1.77, 2.37)	0.0	0.000
Europe	2	0.95 (0.29, 3.13)	90.4	0.928
North America	2	0.85 (0.41, 1.79)	83.1	0.677

### 3.6 Evidence certainty

The GRADE level of evidence for UI risk associated with hysterectomy was very low, as was the evidence for stress and mixed UI. The risk of urge UI was classified as low. For vaginal and laparoscopic hysterectomies, evidence certainty was very low, while abdominal hysterectomy risk was rated as low. The certainty of evidence for UI risk varied across regions, with very low certainty for Europe and North America and low for Asia. GRADE levels for outcomes are summarized in **Table 4**.

**Table 4:** GRADE certainty of evidence.

Outcomes	Exposure	Study of findings		Quality assessment					Certainty of evidence
		No. studies	OR (95%CI)	Study design^*^	Inconsistency^†^	Indirectness	Imprecision	Other consideration	
UI	Hysterectomy	9	1.31 (1.03, 1.66)	serious	serious	not serious	not serious	not serious	Very Low
Stress UI	Hysterectomy	5	1.10 (0.94, 1.21)	serious	serious	not serious	not serious	not serious	Very Low
Urge UI	Hysterectomy	2	1.21 (1.00, 1.46)	serious	not serious	not serious	not serious	not serious	Low
Mixed UI	Hysterectomy	2	1.57 (0.90, 2.74)	serious	serious	not serious	not serious	not serious	Very Low
UI	Abdominal hysterectomy	3	1.21 (1.10, 1.34)	serious	not serious	not serious	not serious	not serious	Low
UI	Vaginal hysterectomy	2	1.24 (0.34, 4.54)	serious	serious	not serious	not serious	not serious	Very Low
UI	Laparoscopy hysterectomy	2	0.70 (0.27, 1.84)	serious	serious	not serious	not serious	not serious	Very Low
UI	Asia	3	2.05 (1.77, 2.37)	serious	not serious	not serious	not serious	not serious	Low
UI	Europe	2	0.95 (0.29, 3.13)	serious	serious	not serious	not serious	not serious	Very Low
UI	North America	2	0.85 (0.41, 1.79)	serious	serious	not serious	not serious	not serious	Very Low

(UI, urinary incontinence, ^*^Downgraded by one level if >25% of participants in this comparison were from studies at high risk of bias. ^†^Downgraded by one level if heterogeneity (I^2^)>50%).

### 3.7 Publication bias

Visual inspection of the funnel plot revealed approximate symmetry regarding the relationship between hysterectomy and UI risk. Egger’s test indicated no significant publication bias in the included studies for headache risk (*P* = 0.797>0.05) (**Supplementary Figure 2**).

## 4 Discussion

### 4.1 Main findings

Our meta-analysis included 12 studies encompassing 146,759 individuals who underwent hysterectomy, providing a detailed exploration of the relationship between hysterectomy and the risk of urinary incontinence (UI). The results indicate a significantly heightened risk of UI in individuals who underwent hysterectomy compared to those who did not, thus suggesting that hysterectomy could serve as an independent risk factor for UI. Noteworthy is the consistency of this association across various subgroup analyses, particularly concerning abdominal hysterectomies and various demographics in Asia, all of which consistently demonstrated a significant link between hysterectomy and an elevated risk of UI.

### 4.2 Interpretation of findings

Previous meta-analyses conducted as early as 2000 examined the connection between hysterectomy and UI risk, drawing on a range of study designs: eight cross-sectional studies, two prospective cohort studies, one case-control study, and one randomized controlled trial (12). In those earlier analyses, women with a history of hysterectomy exhibited increased odds of urinary incontinence, particularly noting a staggering 60% rise in odds for women aged 60 and older, however, odds were not increased for women younger than 60 years.

Our pooled analysis corroborates this historical data, reinforcing the association previously identified while integrating additional insights from 12 studies published post-2008. By analyzing a total of 146,759 individuals who underwent hysterectomy, our findings enrich the understanding of the hysterectomy UI connection. The persistence of this significant association across various geographical regions-Asia, South America, and Oceania-underscores the robustness of the relationship between hysterectomy and the heightened risk of urinary incontinence.

Understanding the physiological implications of hysterectomy is crucial, as it represents a substantial surgical intervention with the potential to instigate chronic or progressive complications, such as urinary incontinence. The surgical procedure may disrupt pelvic nerves and supportive structures, increasing the risk of UI (30, 31). In particular, total hysterectomy, which involves the removal of essential ligamentary support such as the sacral and cardinal ligaments, modifies the anatomical relationships within the pelvis, including the positioning of the bladder and the urethral angle. Such surgical trauma can compromise both urethral support and sphincter innervation, heightening the likelihood of stress urinary incontinence (SUI) (32). Observations of prolonged sacral nerve root latencies immediately following the procedure suggest that the detrimental impacts on continence may not manifest until several years post-surgery. Comparatively, individuals with a prior hysterectomy exhibit significantly lower mean Valsalva leak point pressures, which correlate with an increased risk of severe SUI (11). Additionally, pre-existing factors such as pregnancy, childbirth, pelvic organ surgeries, and urinary tract infections can aggravate bladder inflammation and irritation, further contributing to the risk of UI (33–36). It is noteworthy that many risk factors for UI overlap with those leading to hysterectomy, resulting in some women presenting with incontinence symptoms before the procedure. Therefore, implementing preoperative screening and management strategies for urinary tract infections is vital for reducing the risk of postoperative incontinence, alongside employing surgical techniques designed to minimize pelvic floor trauma and ensuring comprehensive postoperative care. The regional subgroup analysis suggested a higher risk of urinary incontinence among studies conducted in Asia. However, this finding should not be interpreted as definitive evidence of a true regional difference. The number of studies within each regional subgroup was limited, and differences in healthcare systems, surgical practice, indications for hysterectomy, follow-up duration, baseline pelvic floor health, and methods used to ascertain urinary incontinence may have contributed to the observed variation. In addition, cultural differences in symptom reporting and healthcare-seeking behavior may influence the recorded prevalence of urinary incontinence. Therefore, the Asian subgroup finding should be regarded as exploratory and hypothesis-generating.

### 4.3 Strengths and limitations

The strengths of our meta-analysis lie in the incorporation of 12 relevant observational studies, which provides a thorough evaluation of the association between hysterectomy and UI. While prior meta-analyses have examined this connection, our study delivers deeper insights into this complex relationship.

Despite these strengths, several limitations should be acknowledged. First, substantial heterogeneity was observed in the primary analysis, which may limit the interpretability and generalizability of the pooled estimate. Potential sources of heterogeneity include differences in study design, population characteristics, geographical region, age distribution, menopausal status, surgical route, follow-up duration, definitions or ascertainment methods for urinary incontinence, and covariate adjustment strategies. Although subgroup analyses and exploratory meta-regression were performed, these analyses did not fully explain the observed between-study variability. Therefore, the pooled estimate should be interpreted as an average association across clinically and methodologically heterogeneous observational studies rather than as evidence of a uniform effect in all populations.

Second, because all included studies were observational, residual confounding cannot be excluded. Important factors associated with both hysterectomy and urinary incontinence, including parity, age, BMI, menopausal status, pelvic organ prolapse, surgical indication, previous pelvic floor surgery, and baseline urinary symptoms, were not consistently adjusted for across the included studies. Pre-existing urinary incontinence was not uniformly assessed or controlled in all studies. This is clinically relevant because some women may have had urinary symptoms before hysterectomy, and failure to account for baseline incontinence could lead to overestimation or underestimation of the association between hysterectomy and subsequent urinary incontinence. Accordingly, our findings should be interpreted as indicating an association rather than establishing a causal relationship. Future prospective studies with standardized baseline assessment of urinary symptoms and comprehensive adjustment for pelvic floor-related confounders are warranted.

## 5 Conclusions

In conclusion, our meta-analysis indicates a significant association between hysterectomy and the risk of urinary incontinence. The early detection of these conditions is essential for initiating effective intervention strategies and improving long-term patient outcomes. Furthermore, advancements in surgical techniques coupled with adequate postoperative care hold promise for mitigating the risk of urinary incontinence following hysterectomy. Continuous research in this area is necessary to optimize surgical practices and ultimately enhance patient care.

## Data Availability

The original contributions presented in the study are included in the article/**Supplementary Material**. Further inquiries can be directed to the corresponding author.
