# Supplementary material for: Hysterectomy and the risk of urinary incontinence: a systematic review and meta-analysis
Source: Front Urol. 2026 Jun 4;6:1816956. doi: 10.3389/fruro.2026.1816956 (PMC13275263; doi:10.3389/fruro.2026.1816956)
Supplement: Supplementary file 1 [file DataSheet1.docx]

**Supplementary Table 1-3**

**Supplementary Table 1**: PubMed

| No. | Content | Result |
| --- | --- | --- |
| #1 | "Hysterectomy"[Mesh] | 35,622 |
| #2 | Hysterectom*[Title/Abstract] | 47,749 |
| #3 | ("Hysterectomy"[Mesh]) OR (Hysterectom*[Title/Abstract]) | 59,509 |
| #4 | "Urinary Incontinence"[Mesh] | 38,286 |
| #5 | Urinary Incontinence[Title/Abstract] | 32,703 |
| #6 | ("Urinary Incontinence"[Mesh]) OR (Urinary Incontinence[Title/Abstract]) | 49,886 |
| #7 | (("Urinary Incontinence"[Mesh]) OR (Urinary Incontinence[Title/Abstract])) AND (("Hysterectomy"[Mesh]) OR (Hysterectom*[Title/Abstract])) | 1,496 |
| #8 | "Risk"[Mesh] | 1,509,782 |
| #9 | "Risk"[Title/Abstract] | 3,316,196 |
| #10 | "Risk"[MeSH Terms] OR "Risk"[Title/Abstract] | 3,850,222 |
| #11 | ("Risk"[MeSH Terms] OR "Risk"[Title/Abstract]) AND (("Urinary Incontinence"[MeSH Terms] OR "Urinary Incontinence"[Title/Abstract]) AND ("Hysterectomy"[MeSH Terms] OR "hysterectom*"[Title/Abstract])) | 405 |

**Supplementary Table 2:** Embase

| No. | Content | Result |
| --- | --- | --- |
| #1 | 'hysterectomy'/exp | 107,851 |
| #2 | hysterectom*:ab,ti | 78,523 |
| #3 | #1 OR #2 | 119,721 |
| #4 | 'urine incontinence'/exp | 103,505 |
| #5 | 'urinary incontinence':ab,ti | 52,826 |
| #6 | #4 OR #5 | 108,913 |
| #7 | 'risk'/exp | 3,621,111 |
| #8 | risk:ab,ti | 4,859,227 |
| #9 | #7 OR #8 | 5,898,464 |
| #10 | #3 AND #6 AND #9 | 1,483 |

**Supplementary Table 3**: Cochrane Library

| No. | Content | Result |
| --- | --- | --- |
| #1 | MeSH descriptor: [Hysterectomy] explode all trees | 2436 |
| #2 | (Hysterectom*):ti,ab,kw | 9829 |
| #3 | #1 OR #2 | 9831 |
| #4 | MeSH descriptor: [Urinary Incontinence] explode all trees | 3553 |
| #5 | (Urinary Incontinence):ti,ab,kw | 9726 |
| #6 | #4 OR #5 | 9726 |
| #7 | MeSH descriptor: [Risk] explode all trees | 56308 |
| #8 | (Risk):ti,ab,kw | 329481 |
| #9 | #7 OR #8 | 333341 |
| #10 | #3 AND #6 AND #9 | 92 |

**Supplementary Table 4** The excluded studies

|  | **Author** | **Year** | **Title** | Reason for excluded |
| --- | --- | --- | --- | --- |
| 1 | Qian X | 2024 | Incidence and risk factors of stress urinary incontinence after laparoscopic hysterectomy | Incidence(%),not OR/HR/RR |
| 2 | Miranne JM | 2024 | Prevalence of urinary incontinence and prolapse after hysterectomy for benign disease versus gynecologic malignancy | Not meet the eligibility criteria |
| 3 | Lee JH | 2023 | Hysterectomy for uterine fibroids and stress urinary incontinence surgery: A nationwide cohort study | Conference abstracts |
| 4 | Lee JH | 2023 | Risk of Anti-Urinary Incontinence Surgery After Hysterectomy for Uterine Fibroids: A Nationwide Cohort Study | Conference abstracts |
| 5 | Christoffersen NM | 2023 | Increased risk of stress urinary incontinence surgery after hysterectomy for benign indication-a population-based cohort study | Not meet the eligibility criteria |
| 6 | Christoffersen NM | 2022 | Increased Risk of Stress-urinary-incontinence Surgery after Hysterectomy - A Population-based Cohort Study | Conference abstracts |
| 7 | Husby KR | 2022 | Stress Urinary Incontinence Following Manchester Procedure and Vaginal Hysterectomy: A Nationwide Cohort Study | Conference abstracts |
| 8 | Tulokas S | 2022 | Efect of hysterectomy on re-operation for stress urinary incontinence: 10 year follow-up | Not meet the eligibility criteria |
| 9 | Tulokas S | 2022 | Stress urinary incontinence after hysterectomy: a 10-year national follow-up study | Not meet the eligibility criteria |
| 10 | Patel UJ | 2020 | Updating the prevalence of urinary incontinence in adult women using 2015-2016 data from a national population-based survey | Conference abstracts |
| 11 | O'Shea BJ | 2019 | Urinary incontinence symptoms after hysterectomy for endometrial adenocarcinoma | Conference abstracts |
| 12 | Christiansen UJ | 2017 | Hysterectomy is not associated with de-novo urinary incontinence: A ten-year cohort study | Not meet the eligibility criteria |
| 13 | Bohlin KS | 2016 | Factors influencing the incidence and remission of urinary incontinence after hysterectomy | Not meet the eligibility criteria |
| 14 | Skorupska KA | 2016 | Urinary incontinence after hysterectomy- does type of surgery matter? | Not meet the eligibility criteria |
| 15 | Andersen LL | 2015 | Lower urinary tract symptoms after subtotal versus total abdominal hysterectomy: exploratory analyses from a randomized clinical trial with a 14-year follow-up | Not meet the eligibility criteria |
| 16 | Chang KM | 2014 | Risk factors for urinary incontinence among women aged 60 or over with hypertension in Taiwan | Not meet the eligibility criteria |
| 17 | Kudish BI | 2013 | The effect of hysterectomy, with and without BSO, on urinary incontinence | Conference abstracts |
| 18 | Singh U | 2013 | Prevalence and risk factors of urinary incontinence in Indian women: A hospital-based survey | Not meet the eligibility criteria |
| 19 | Forsgren C | 2012 | Vaginal hysterectomy and risk of pelvic organ prolapse and stress urinary incontinence surgery | Duplicate records |
| 20 | Forsgren C | 2012 | Vaginal hysterectomy and risk of pelvic organ prolapse and stress urinary incontinence surgery | Not meet the eligibility criteria |
| 21 | Dällenbach P | 2011 | Incidence and risk factors for reoperation after surgically managed urinary incontinence: A nested case control study | Conference abstracts |
| 22 | Lakeman MM | 2011 | Predicting the development of stress urinary incontinence 3 years after hysterectomy | Not meet the eligibility criteria |
| 23 | Hsieh CH | 2008 | Risk factors for urinary incontinence in Taiwanese women aged 20-59 years | Not meet the eligibility criteria |
| 24 | Daneshgari F | 2008 | Differences in urinary incontinence between Hispanic and non-Hispanic white women: a population-based study | Not meet the eligibility criteria |
| 25 | Altman D | 2007 | Hysterectomy and risk of stress-urinary-incontinence surgery: nationwide cohort study | Not meet the eligibility criteria |
| 26 | Neumann GA | 2007 | Incidence and remission of urinary incontinence after hysterectomy--a 3-year follow-up study | Not meet the eligibility criteria |
| 27 | Ellström Engh M | 2006 | Hysterectomy and incontinence: A study from the Swedish national register for gynecological surgery | Duplicate records |
| 28 | Gustafsson C | 2006 | Urinary incontinence after hysterectomy-three-year observational study | Duplicate records |
| 29 | Engh MA | 2006 | Hysterectomy and incontinence: a study from the Swedish national register for gynecological surgery | Not meet the eligibility criteria |
| 30 | Gustafsson C | 2006 | Urinary incontinence after hysterectomy--three-year observational study | Not meet the eligibility criteria |
| 31 | Huang AJ | 2006 | Urinary incontinence and pelvic floor dysfunction in Asian-American women | Not meet the eligibility criteria |
| 32 | De Tayrac R | 2004 | Risk of urge and stress urinary incontinence at long-term follow-up after vaginal hysterectomy | Retracted article |
| 33 | Gimbel H | 2004 | Subtotal hysterectomy increased the risk of urinary incontinence compared to total hysterectomy | Short Survey |
| 34 | Buchsbaum GM | 2002 | Prevalence of urinary incontinence and associated risk factors in a cohort of nuns | Not meet the eligibility criteria |
| 35 | Jueng-Anuwat P | 2001 | Risk factors for stress urinary incontinence in middle aged and elderly Thai women | Full text unavailable |

**Supplementary Table 5** Details of NOS

| Study | Year | Selection | Comparability | Outcome | Total |
| --- | --- | --- | --- | --- | --- |
| Salo H | 2024 | ★★★ | ★★ | ★★ | 7 |
| Yuk JS | 2023 | ★★★ | ★ | ★★ | 6 |
| Li PC | 2019 | ★★★ | ★★ | ★★ | 7 |
| Kudish BI | 2014 | ★★★ |  | ★★ | 5 |
| Kirss F | 2013 | ★★ |  | ★★ | 4 |
| Byles J | 2009 | ★★★ |  | ★★ | 5 |
| Ham E | 2009 | ★★ |  | ★★ | 4 |
| Miller JJ | 2008 | ★★ |  | ★★ | 4 |
| Minassian VA | 2008 | ★★ |  | ★★ | 4 |

**Supplementary Table 6** Details of AHRQ

| Study | Year | AHRQ score |
| --- | --- | --- |
| Juliato CR | 2017 | 5 |
| Linde JM | 2017 | 1 |
| Barghouti FF | 2013 | 5 |

**Supplementary Table 7** Sensitivity analyses for UI risk of hysterectomy

|  |  | **OR** | **95% CI** | **I**2**/%** | ***P* value** |
| --- | --- | --- | --- | --- | --- |
|  | **Total** | 1.31 | 1.03-1.66 | 88.5 | 0.029 |
|  | **Excluded study** |  |  |  |  |
| 1 | Salo H 2024 | 1.42 | 1.12-1.80 | 88.1 | 0.003 |
| 2 | Li PC 2019 | 1.21 | 0.99-1.48 | 75.6 | 0.068 |
| 3 | Juliato CR 2017 | 1.29 | 0.99-1.68 | 89.9 | 0.059 |
| 4 | Kudish BI 2014 | 1.32 | 0.96-1.81 | 89.6 | 0.091 |
| 5 | Kirss F 2013 | 1.27 | 0.98-1.63 | 89.7 | 0.069 |
| 6 | Barghouti FF 2013 | 1.25 | 0.98-1.61 | 89.6 | 0.076 |
| 7 | Byles J 2009 | 1.33 | 0.98-1.81 | 87.8 | 0.068 |
| 8 | Ham E 2009 | 1.24 | 0.97-1.58 | 89.2 | 0.086 |
| 9 | Miller JJ 2008 | 1.40 | 1.10-1.79 | 88.7 | 0.006 |


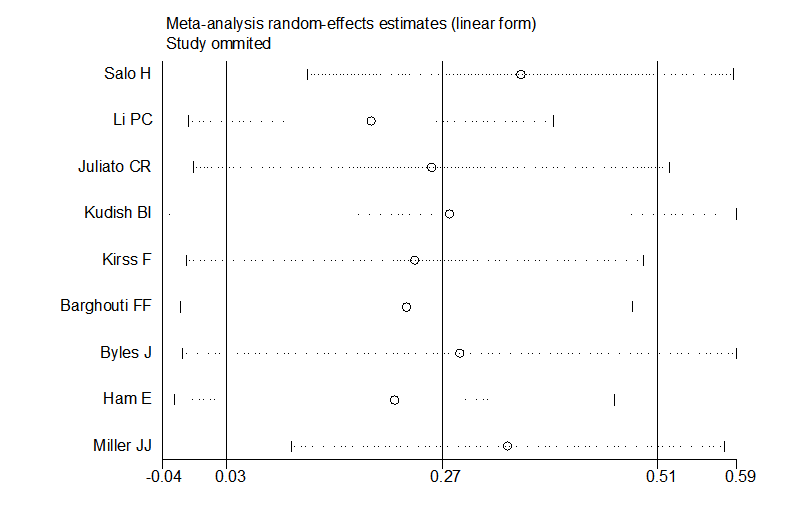
**Supplementary Figure1** Sensitivity analyses for UI risk of hysterectomy


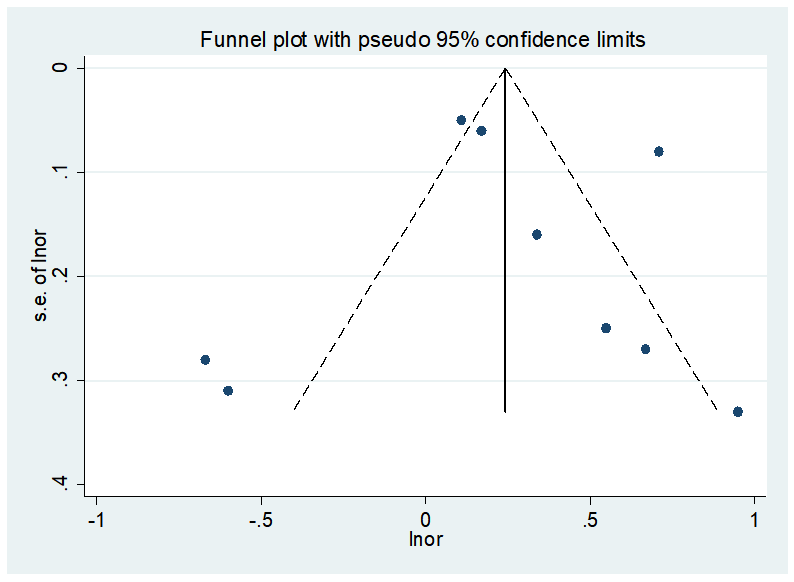
**Supplementary Figure 2** Publication bias for UI risk of hysterectomy
